# Transmission of cell-associated human cytomegalovirus isolates between various cell types using polymorphonuclear leukocytes as a vehicle

**DOI:** 10.1007/s00430-021-00713-6

**Published:** 2021-06-06

**Authors:** Berenike Braun, Christian Sinzger

**Affiliations:** Institute for Virology, Ulm University Medical Center, Ulm, Germany

**Keywords:** HCMV, Clinical isolates, Cell-associated spread, Hematogenous dissemination, PMNs, PDGFRα-peptides

## Abstract

Polymorphonuclear leukocytes (PMNs) are regarded as vehicles for the hematogenous dissemination of human cytomegalovirus (HCMV). In cell culture, this concept has been validated with cell-free laboratory strains but not yet with clinical HCMV isolates that grow strictly cell-associated. We, therefore, aimed to evaluate whether PMNs can also transmit such isolates from initially infected fibroblasts to other cell types, which might further clarify the role of PMNs in HCMV dissemination and provide a model to search for potential inhibitors. PMNs, which have been isolated from HCMV-seronegative individuals, were added for 3 h to fibroblasts infected with recent cell-associated HCMV isolates, then removed and transferred to various recipient cell cultures. The transfer efficiency in the recipient cultures was evaluated by immunofluorescence staining of viral immediate early antigens. Soluble derivatives of the cellular HCMV entry receptor PDGFRα were analyzed for their potential to interfere with this transfer. All of five tested HCMV isolates could be transferred to fibroblasts, endothelial and epithelial cells with transfer rates ranging from 2 to 9%, and the transferred viruses could spread focally in these recipient cells within 1 week. The PDGFRα-derived peptides IK40 and GT40 reduced transfer by 40 and 70% when added during the uptake step. However, when added during the transfer step, only IK40 was effective, inhibiting transmission by 20% on endothelial cells and 50–60% on epithelial cells and fibroblasts. These findings further corroborate the assumption of cell-associated HCMV dissemination by PMNs and demonstrate that it is possible to inhibit this transmission mode.

## Introduction

The human cytomegalovirus (HCMV) belongs to the family of herpesviruses and is widespread in the population. While most immunocompetent people do not even notice their primary infection [1], this virus can be life threatening for immunocompromised patients like transplant recipients and is still the leading viral cause of congenital infections [2–5]. In these situations, a variety of organs can become infected [6, 7], probably via the bloodstream where the infectious virus is found almost exclusively in the cellular fraction [8–10]. In particular, circulating cytomegalic endothelial cells have the potential to carry HCMV infection to distant sites of the vascular system, infected monocytes may infiltrate tissues and spread the virus after differentiation into macrophages, and finally polymorphonuclear leukocytes (PMNs) are considered vehicles for the uptake and dissemination of infectious virions, while not being productively infected themselves [11–14]. Actually, PMNs are the cellular blood fraction in which the highest viral titers have been observed in HCMV-infected patients [11, 15], and the detection of the viral tegument protein pp65 in the nucleus of PMNs is exploited as a diagnostic “antigenemia” assay [16]. Transmission of HCMV via leukocytes has also been addressed in cell culture. Monocytes, which are also regarded nonpermissive for a productive replication cycle, could transmit an endothelial cell-adapted HCMV strain between infected and uninfected monolayer cultures [17]. PMNs were repeatedly shown to take up HCMV from infected monolayer cultures as indicated be the detection of pp65 in the nucleus [18–21] and were also reported to transfer infectious virus to uninfected monolayers [22]. Although low-passage isolates were included in these analyses, formal proof was missing that they were still growing in a cell-associated manner. Hence, it is still unclear whether PMNs can also take up HCMV from infected cells that do not release infectious progeny. After isolation from clinical specimens, HCMV initially grows in a strictly cell-associated manner, releasing cell-free infectivity only after cell culture adaptation associated with genetic alterations [23–28]. While attachment and penetration of cell-free virus into target cells can be inhibited by soluble derivatives of the cellular HCMV receptor platelet-derived growth factor receptor alpha (PDGFRα) [29–31], it is unclear whether any of these derivatives can also inhibit isolates that spread strictly cell-associated. Mechanistically, their mode of action resembles inhibition by neutralizing antibodies (nAbs), which can bind to epitopes on the surface of virions, prevent their entry into the target cell, and thereby completely inhibit infection with cell-free HCMV [32]. In contrast, focal spread of cell culture-adapted HCMV strains is only partially reduced by nAbs and PDGFRα-Fc [30, 33], and the antibody-resistant part of focal spread has been interpreted as “cell-associated” transmission or “cell-to-cell” spread. This assumption is supported by the findings that nAbs do not reduce focal spread under conditions where cell-free virus is captured by an agarose overlay [34] and that strictly cell-associated HCMV isolates are completely resistant to antibodies [33, 35]. One explanation is that nAbs and PDGFRα-Fc are too large and are hence excluded from sites of virus transmission. Smaller 40-mer fragments of the extracellular domain of PDGFRα were also identified as entry inhibitors [29], and it is tempting to assume that they might have an impact on cell-associated transmission.

Aim of this study was to test whether PMNs can transfer recent HCMV isolates that grow strictly cell-associated from infected to uninfected cell cultures, which could serve as a cell culture model for PMN-mediated virus dissemination in vivo and enable to evaluate the effect of entry inhibitors on this transmission mode. Furthermore, use of PMNs as vehicles might facilitate the transfer of recent cell-associated HCMV isolates from fibroblast cultures to endothelial or epithelial cells.

## Materials and methods

### Cells and viruses

Primary human foreskin fibroblasts (HFFs) were cultivated in MEM with GlutaMax (Gibco) supplemented with 5% fetal bovine serum (FBS, PAN Biotech), 0.5 ng/ml basic fibroblast growth factor (tebu bio) and 100 µg/ml gentamicin. For experiments, cells were cultured in medium without the basic fibroblast growth factor (denoted by MEM5). Conditionally immortalized human endothelial cells (HEC-LTTs) [36, 37] were cultivated on cell culture flasks coated with gelatin in human endothelial cell growth medium (PromoCell) supplemented with 2 µg/ml doxycycline. For experiments, HEC-LTTs were seeded in medium without doxycycline. Adult retinal pigment epithelial cells (ARPE-19) were cultured in DMEM/F-12 with GlutaMax (Gibco) supplemented with 5% FBS and 100 µg/ml gentamicin.

Recent clinical HCMV isolates, provided by the diagnostic laboratory of the Institute for Virology in Ulm, were propagated to high infection rates of about 50–70% in HFFs and then stored in aliquots at − 80 °C until used in experiments. HFFs infected with the isolate-like strain Merlin-pAL1502 [27] at an infection multiplicity of 0.05 were used 6 days post-infection (d p. i.) to achieve an infection rate similar to that of clinical isolates. For each individual PMN-mediated transfer experiment, clinical isolates and Merlin-pAL1502 were analyzed regarding their cell-associated phenotype. For that, 15,000 HFFs/well were incubated overnight in 96-well plates with the cell-free supernatant that had been centrifuged at 2790 × *g* for 10 min, fixed the next day, and stained for viral immediate early (IE) antigen (Ag) by indirect immunofluorescence. The donor cultures were considered to grow strictly cell-associated if no more than ten IE Ag-positive cells per well were detected.

PMNs were isolated from EDTA blood of HCMV-seronegative donors via Polymorphprep (Progen) according to the manufacturer’s instructions, including lysis of contaminating erythrocytes with NH_4_Cl for 5 min on ice, and were immediately used for transfer experiments.

### Transfer of cell-associated HCMV via PMNs

HFFs infected with five different recent clinical isolates or Merlin-pAL1502 in 6-well plates served as donor cultures for PMN-mediated transfer of cell-associated HCMV. Uninfected HFFs, HEC-LTTs or ARPE-19 cells seeded on gelatin-coated 96-well plates were used as recipient cultures. PMNs were incubated with the donor cultures at a ratio of 10:1 for 3 h at 37 °C in MEM5 and then recollected, carefully avoiding detachment of the donor culture layer. Part of the recollected PMNs were prepared for immunofluorescence staining of viral pp65 Ag by centrifugation in a StatSpin Cytofuge for 5 min at 395 × *g*, followed by drying for 1 h in front of a blower. The remaining fraction of PMNs was again distributed to the recipient cultures in a 10:1 ratio. After 3 h of incubation at 37 °C, supernatants were removed, the cell type-specific medium was added and cultures were incubated overnight at 37 °C. On the next day, cells were fixed and stained for viral pp65 or IE Ag via indirect immunofluorescence. Cell nuclei were counterstained with 4′,6-diamidino-2-phenylindole (DAPI). Transfer efficiencies were determined as the fraction of IE Ag-positive cells compared to the total cell number.

### Propagation of clinical isolates in HEC-LTTs and ARPE-19 cells upon transfer

To analyze whether clinical isolates can be propagated in recipient cell cultures after transfer, PMN-mediated transfer was performed as previously described with four different clinical isolates using HFFs, HEC-LTTs and ARPE-19 cells as recipients. Each transfer was performed in duplicates. One of the replicate cultures was fixed the next day, stained for viral IE Ag by indirect immunofluorescence, and transfer efficiencies were determined as previously described. For the remaining replicate culture, the medium was refreshed the next day, and after 6 days, the culture was fixed, stained for viral IE Ag by indirect immunofluorescence, and the number of HCMV-infected cells per focus was counted. Foci were evaluated as an accumulation of at least 3 IE Ag-positive cells in direct surrounding.

### Evaluating the effects of HCMV entry inhibitors on PMN-mediated spread

HCMV-specific hyperimmunoglobulin (Cytotect, CP Biotest), recombinant PDGFRα-Fc (R&D Systems) and the PDGFRα-derived 40-mer peptides GT40 and IK40 [29] (Phtdpeptides) were evaluated regarding their inhibitory effect on PMN-mediated virus transmission. Experiments were conducted as described before with the following modification: inhibitors were added during incubation of PMNs with either donor cultures (“uptake”) or recipient cultures (“transfer”). The antiviral agents were each diluted in MEM5 to achieve complete inhibition of cell-free virus (at least 10 × EC50). As a negative control for peptide treatments, 0.4% dimethyl sulfoxide solution was applied, resembling the concentration of this solvent in the peptide assays. For the other antiviral agents, MEM5 was used as control. The respective concentrations were 0.5 mg/ml for hyperimmunoglobulin, 120 ng/ml for PDGFRα-Fc and 0.45 mg/ml for GT40 and IK40 (EC50 of GT40 = 0.009 mg/ml, EC50 of IK40 = 0.045 mg/ml). To exclude the possibility that the results are primarily determined by the amount of substance used, both peptides were used at the same concentration. Evaluation of transfer efficiencies was done as described before. For the mode of action analysis of the peptide IK40, µclear 96-well plates were used to allow detection of single viral particles.

### Indirect immunofluorescence

On the day after PMN-mediated transfer, cells were fixed with 80% acetone for 5 min at room temperature (RT) and washed thrice with phosphate buffered saline (PBS). Primary antibodies were applied for 90 min followed by washing three times with PBS. Secondary antibodies were applied for 60 min at 37 °C, again followed by washing with PBS. Cell nuclei were counterstained with DAPI (Sigma) for 8 min at RT. Viral IE Ag was detected with the monoclonal mouse antibody E13 (Argene) and Cy3-goat-anti-mouse Ig F(ab´)_2_ (Jackson ImmunoResearch) or alternatively Alexa488-goat-anti-mouse Ig F(ab´)_2_ (Thermofisher Scientific), if also viral particles were detected. Staining of IE Ag resulted in a nuclear fluorescent pattern. Viral pp65 was detected using monoclonal mouse antibody 28–77 (kindly provided by W. Britt, Birmingham, AL, USA) and Alexa488-goat-anti-mouse Ig F(ab´)_2_ and resulted in a nuclear fluorescent pattern. For staining of viral particles, monoclonal mouse antibody 36–14 [38] and Alexa555-goat-anti-mouse Ig F(ab´)_2_ (Thermofisher Scientific) were used to detect the capsid-associated tegument protein pp150, and this staining resulted in dot-like fluorescence.

For detection of pp65 in cytocentrifuged PMN preparations, the dried cytospots were fixed with 1% paraformaldehyde (Sigma-Aldrich), permeabilized with 10% sucrose (Sigma-Aldrich), 1% FBS and 0.5% Nonidet P40 (Sigma-Aldrich) for 10 min at RT, and blocked for 30 min at 37 °C with 5% FBS. Detection of viral pp65 Ag was then performed using the previously described antibodies, each applied for 45 min at 37 °C. Nuclei were counterstained with DAPI for 30 s at RT.

### Structural visualization of PDGFRα-derived peptides

The cryoEM structure of the trimeric HCMV gH/gL/gO complex bound to human PDGFRα and neutralizing fabs 13H11 and MSL-109 (PDB:7LBF [39]) was used to visualize the positions of the inhibitory peptides GT40 and IK40. The image was created in UCSF Chimera, which was developed by the Resource for Biocomputing, Visualization, and Informatics at the University of California, San Francisco [40].

### Statistical analysis

Statistical analyses were done using the build-in data analysis functions of Microsoft Excel. Datasets were first evaluated using a one-way ANOVA regarding differences between various conditions. If the ANOVA indicated significant differences within the dataset, a post hoc analysis was added, comparing each condition individually with the untreated control by unpaired two-sided *t* tests.

## Results

While the HCMV-antigenemia phenomenon has already been simulated in cell culture, demonstrating uptake of pp65 Ag by PMNs from monolayer cultures infected with HCMV laboratory strains [18, 19, 21, 41], it has not yet been formally demonstrated whether PMNs can also take up strictly cell-associated HCMV isolates from infected cultures and transfer them to uninfected cell layers. We, therefore, aimed to establish a cell culture model for all steps of PMN-mediated dissemination of HCMV, including (1) uptake of cell-associated isolates by PMNs, (2) transfer to various target cell types and (3) subsequent propagation of the isolates in these recipient cultures. This model should then be applied to test the effect of various entry inhibitors on this transmission mode.

### Cell-associated isolates are transmitted via PMNs onto various recipient cell types and subsequently replicate in these cells

**Fig. 1:**
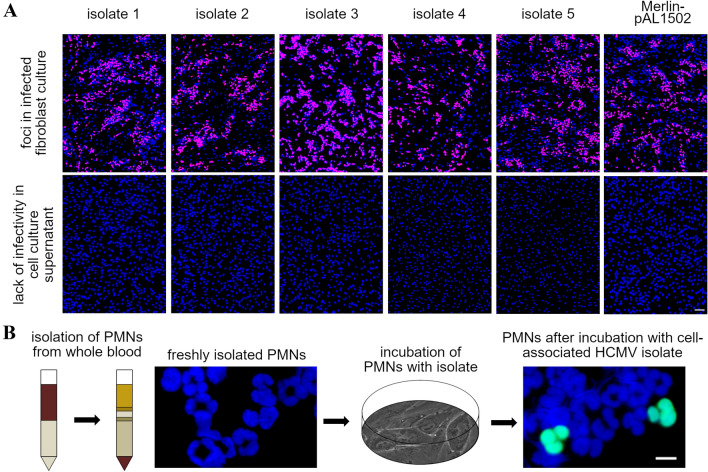
PMNs take up cell-associated HCMV isolates from infected HFFs. **A** Uninfected HFFs were either co-cultured with HFFs infected with 5 different clinical isolates or infected with Merlin-pAL1502. These cultures were used as donor cultures 4–7 d p. i. (upper panel). Their cell culture supernatants were analyzed for cell-free infectivity on uninfected HFFs on the day of each transfer experiment (lower panel). Cells were fixed on the next day and stained via indirect immunofluorescence for viral IE Ag (pink nuclei). Cell nuclei were counterstained with DAPI (blue nuclei). Scale bar = 100 µm. **B** PMNs isolated from fresh blood samples of HCMV-seronegative donors were either directly stained for viral pp65 Ag (green nuclei) or after incubation with the HCMV donor cultures for 3 h at 37 °C. Cell nuclei were counterstained with DAPI (blue nuclei)

**Fig. 2:**
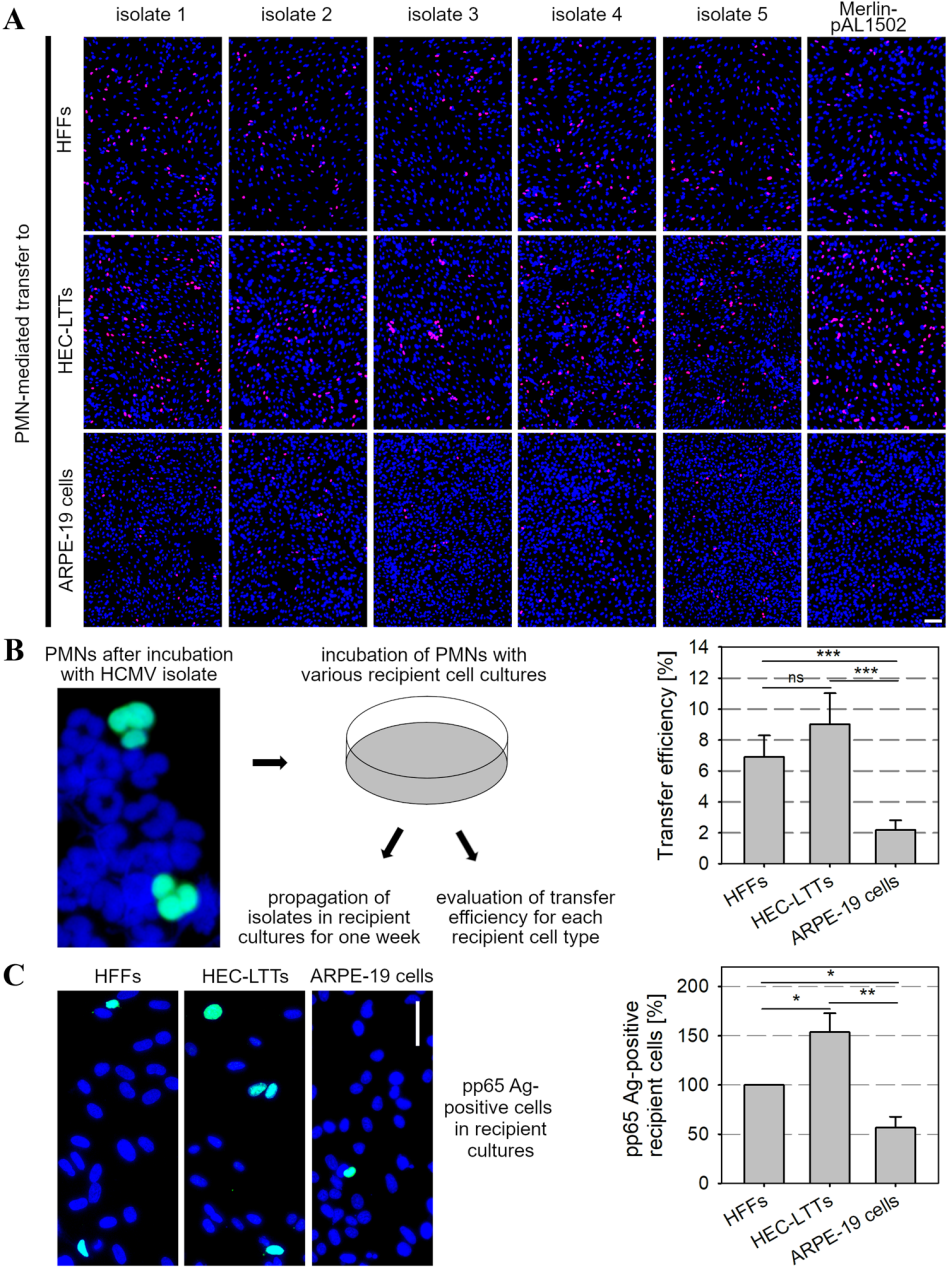
PMNs transfer cell-associated HCMV isolates with different susceptibility to various recipient cell cultures. **A** PMNs were recollected after incubation with the donor cultures for 3 h at 37 °C and applied onto uninfected HFFs, HEC-LTTs or ARPE-19 cells. After 3 h, PMNs were removed. On the next day, recipient cells were fixed and stained for viral IE Ag via indirect immunofluorescence (pink nuclei). Cell nuclei were counterstained with DAPI (blue nuclei). Scale bar = 100 µm. **B** Schematic of the transfer procedure and determination of transfer efficiencies onto the recipient cultures as the ratio of infected cells to the total cell number. Error bars represent the standard error of the mean (SEM) of 11 individual experiments, asterisks indicate significant differences (***p value < 0.001). **C** PMN-mediated transfer was performed with 3 different clinical isolates. On the next day, recipient cells were fixed and stained for viral pp65 Ag via indirect immunofluorescence (green nuclei). Cell nuclei were counterstained with DAPI (blue nuclei). Scale bar = 50 µm. The percentage of pp65 Ag-positive recipient cells was determined as a ratio of total cell number. Error bars represent the SEM of 3 individual experiments, asterisks indicate significant differences (*p value < 0.05; **p value < 0.01)

HFFs infected with five different clinical isolates or the isolate-like strain Merlin-pAL1502 served as donor cultures for the following transfer experiments. To test whether HCMV was truly cell-associated in these donor cultures, supernatants were harvested, clarified from cells and debris and added to uninfected HFFs for an overnight incubation and subsequent immunofluorescence staining of viral IE Ag. Only if the number of cells positive for IE Ag was below the cutoff of 10 per 15,000 cells, the respective donor cultures were used for transfer experiments (Fig. 1A). For the transfer, PMNs were extracted from fresh blood samples of HCMV-seronegative donors via density centrifugation. Immediately after they have been harvested from the gradient, they were applied to the donor cultures and recollected after 3 h of incubation. To visualize an uptake of HCMV to the PMNs, a fraction of cells was stained for viral pp65 Ag via immunofluorescence. Strong nuclear pp65 signals were detected in a minor percentage of PMNs incubated with an HCMV donor culture (Fig. 1B), whereas untreated PMNs were always negative, confirming that the granulocytes indeed take up virus from the donor culture. The remaining PMNs were incubated for 3 h with various uninfected recipient cell types: HFFs, HEC-LTTs or ARPE-19 cells. The recipient cells were fixed, stained for viral IE Ag via immunofluorescence and counterstained with DAPI on the next day. In all recipient cultures, cells expressing IE Ag were detected (Fig. 2A). Transfer efficiency was calculated as the ratio of cells positive for viral IE Ag to the total cell number, and this was 2% on ARPE-19 cells, 7% on HFFs, and 9% on HEC-LTTs (Fig. 2B). The difference between epithelial cells versus fibroblasts (*p* value < 0.001) and endothelial cells (*p* value < 0.001) was significant. Next, we wanted to check whether the different infection rates of the recipient cell types could indeed be explained by different transfer efficiencies or were rather due to steps after particle transfer. For this purpose, we additionally analyzed the recipient cells the day after transfer by indirect immunofluorescence for pp65 Ag, which can serve as a marker for successful penetration (Fig. 2C). The number of pp65 Ag-positive cells was determined in relation to the total cell number, and as with IE Ag evaluation, there were significant differences between the recipient cell types. Compared to the number of pp65 Ag-positive HFFs, about 50% more positive cells were detected in HEC-LTT cultures (*p* value < 0.05) and about 50% fewer in ARPE-19 cell cultures (*p* value < 0.05).

**Fig. 3:**
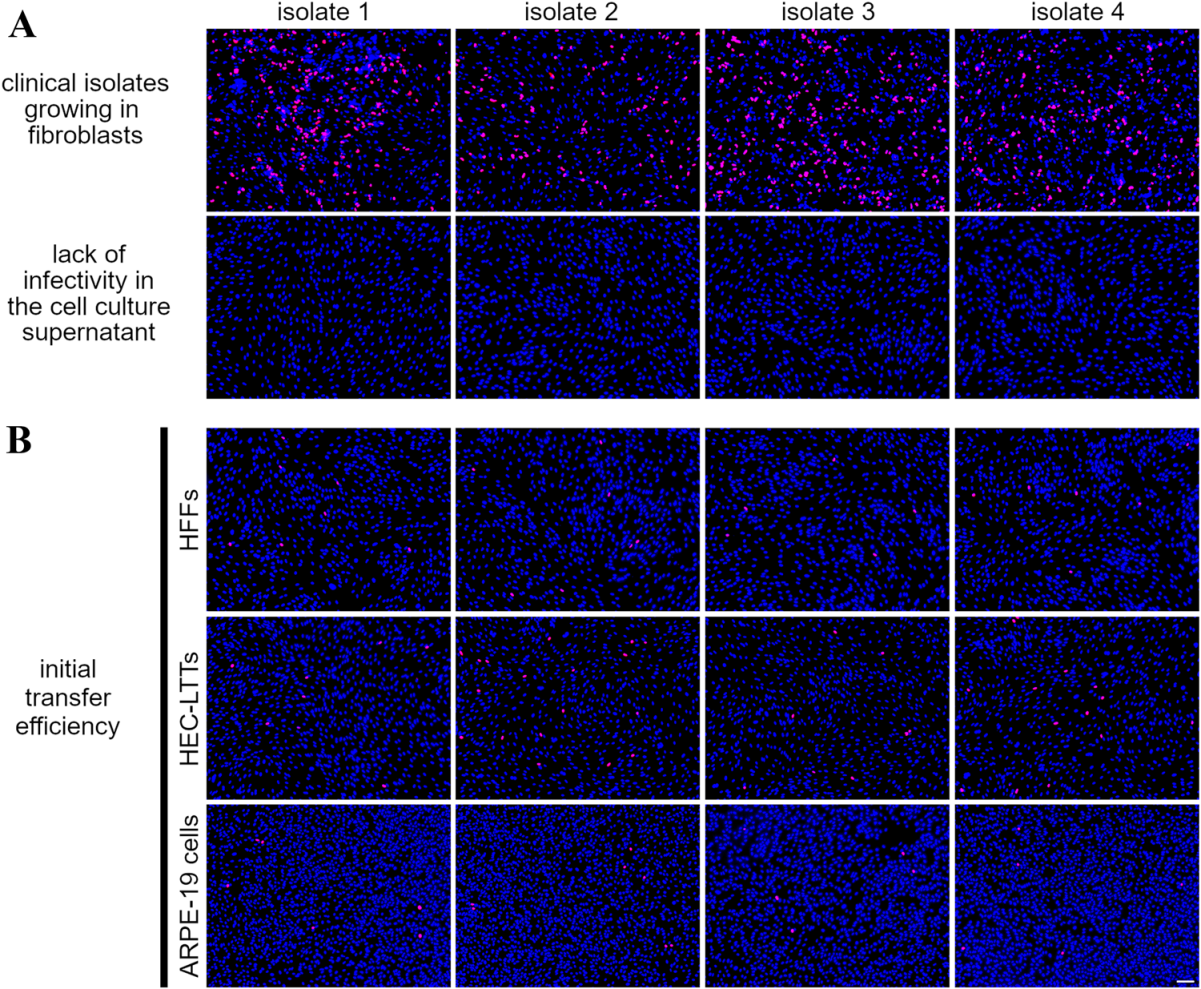

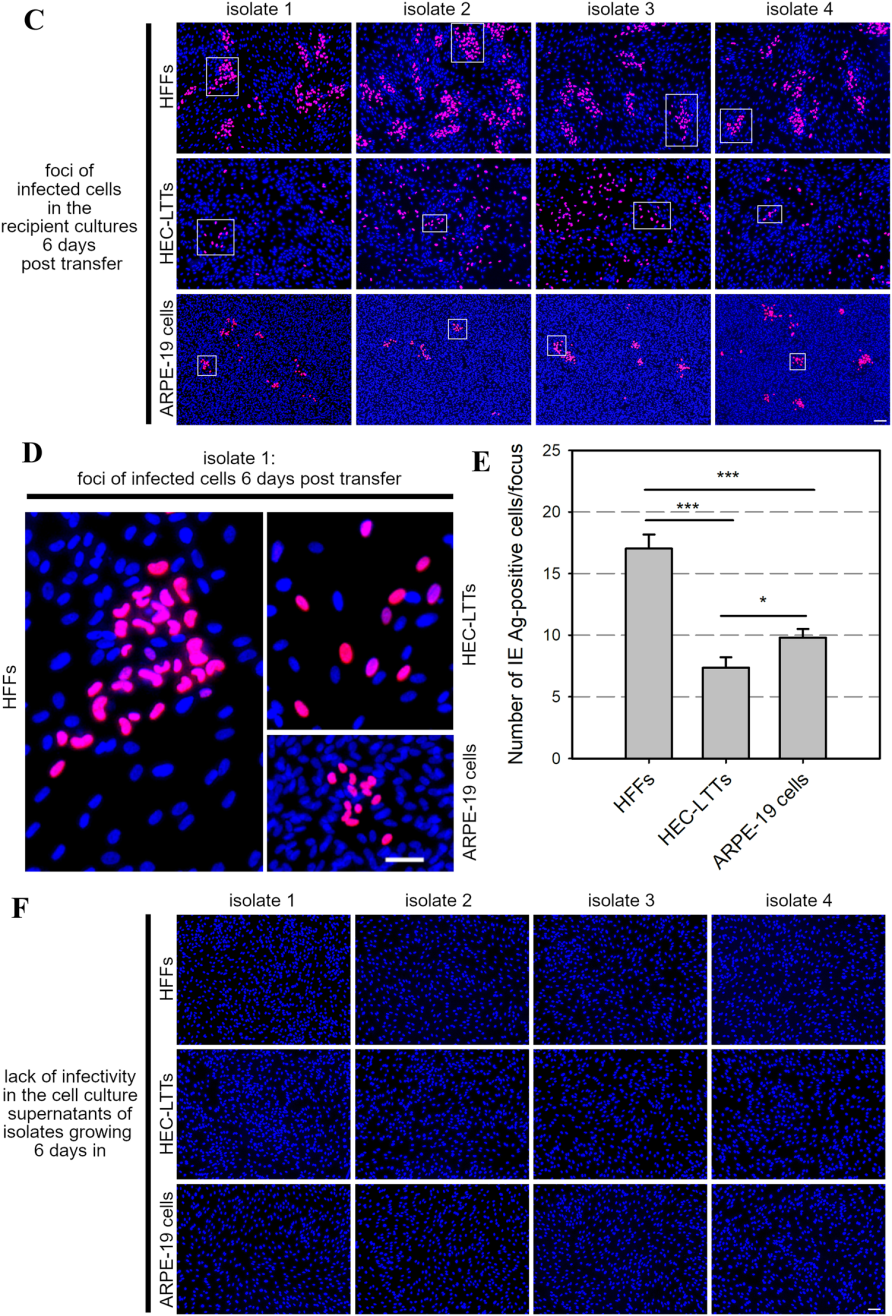
Clinical isolates grow in a focal fashion in the recipient cultures after being transferred from infected HFFs via PMNs. **A** Uninfected HFFs were co-cultured with HFFs infected with 4 different clinical isolates. After 2 days, these cultures were used as donor cultures for PMN-mediated transmission. The cell culture supernatants of the isolates were analyzed for cell-free infectivity on uninfected HFFs at the day of the transfer experiment. PMNs isolated from fresh blood samples of HCMV-seronegative donors were incubated with the donor cultures for 3 h at 37 °C, then recollected and incubated with uninfected HFFs, HEC-LTTs or ARPE-19 cells. After 3 h at 37 °C, PMNs were removed. **B** Recipient cell cultures were fixed on the next day and stained for viral IE Ag via indirect immunofluorescence (pink nuclei). Cell nuclei were counterstained with DAPI (blue nuclei). Scale bar = 100 µm. **C** Replica cultures were incubated for 6 days to allow growth of the isolates in the recipient cells and then fixed and stained for viral IE Ag (pink nuclei). Cell nuclei were counterstained with DAPI (blue nuclei). Scale bar = 100 µm. In each immunofluorescence image, one exemplary focus is framed. **D** All exemplary frames of isolate 1 are shown magnified. Scale bar = 50 µm. **E** The recipient cultures were evaluated for the number of IE Ag-positive cells per focus 6 days post-transfer. For each cell type, 70 foci were analyzed. Error bars represent the standard error of the mean of 4 individual experiments, asterisks indicate significant differences (*p < 0.05; ***p < 0.001). **F** Cell culture supernatants of isolates growing for 6 days in the recipient cultures were analyzed for cell-free infectivity on uninfected HFFs. Cell nuclei were counterstained with DAPI (blue nuclei). Scale bar = 100 µm

We then analyzed whether isolates would replicate in the recipient cell cultures upon successful PMN-mediated transfer from infected fibroblast cultures. Four different clinical isolates growing in HFFs were used as donor cultures and their cell culture supernatants were evaluated regarding cell-free infectivity on uninfected HFFs (Fig. 3A). The isolates were transferred via PMNs as described before to HFFs, HEC-LTTs and ARPE-19 cells. Each recipient cell type was infected in duplicate. One replica was fixed and stained for IE Ag via immunofluorescence on the next day as a control whether the initial transfer of the isolate was successful, and again all cultures showed cells positive for viral IE Ag (Fig. 3B). As observed in the previous transfer experiments, the highest transfer efficiencies were detected on HEC-LTTs followed by HFFs and ARPE-19 cells. The remaining replicas of the recipient cultures were further incubated at 37 °C for 6 days to allow growth and spread of the transferred virus. Finally, supernatants were again harvested and controlled regarding the cell-associated phenotype, and cell layers were fixed and stained for viral IE Ag via immunofluorescence. Foci of infected cells were detected in every recipient culture for the four transferred isolates (Fig. 3C). However, the shape of the foci differed between the recipient cell types. All isolates showed clearly defined foci in HFFs and ARPE-19 cells, whereas the foci in HEC-LTTs were less condensed (Fig. 3D). Quantitative differences could be observed when foci of infected cells were compared. The mean focus size of 70 analyzed foci per recipient cell type was determined as 17 for HFFs (maximum 70 cells per focus), 10 for ARPE-19 cells (maximum 27 cells per focus) and 7 for HEC-LTTs (maximum 36 cells per focus) (Fig. 3E). Foci in fibroblasts were hence significantly larger than in endothelial cells and epithelial cells (*p* values < 0.001), and the difference between endothelial and epithelial cells was also significant (*p* value < 0.05). Nevertheless, the absence of IE Ag in HFFs after incubation with the supernatants confirmed that the isolates did not release cell-free infectious virus 6 days after transfer (Fig. 3F). Our results indicate that HCMV isolates grow in different recipient cell types upon PMN-mediated transfer and the cell-associated phenotype of the isolates was maintained during the transfer procedure.

### PDGFRα-derived peptides inhibit PMN-mediated transmission of HCMV isolates

**Fig. 4:**
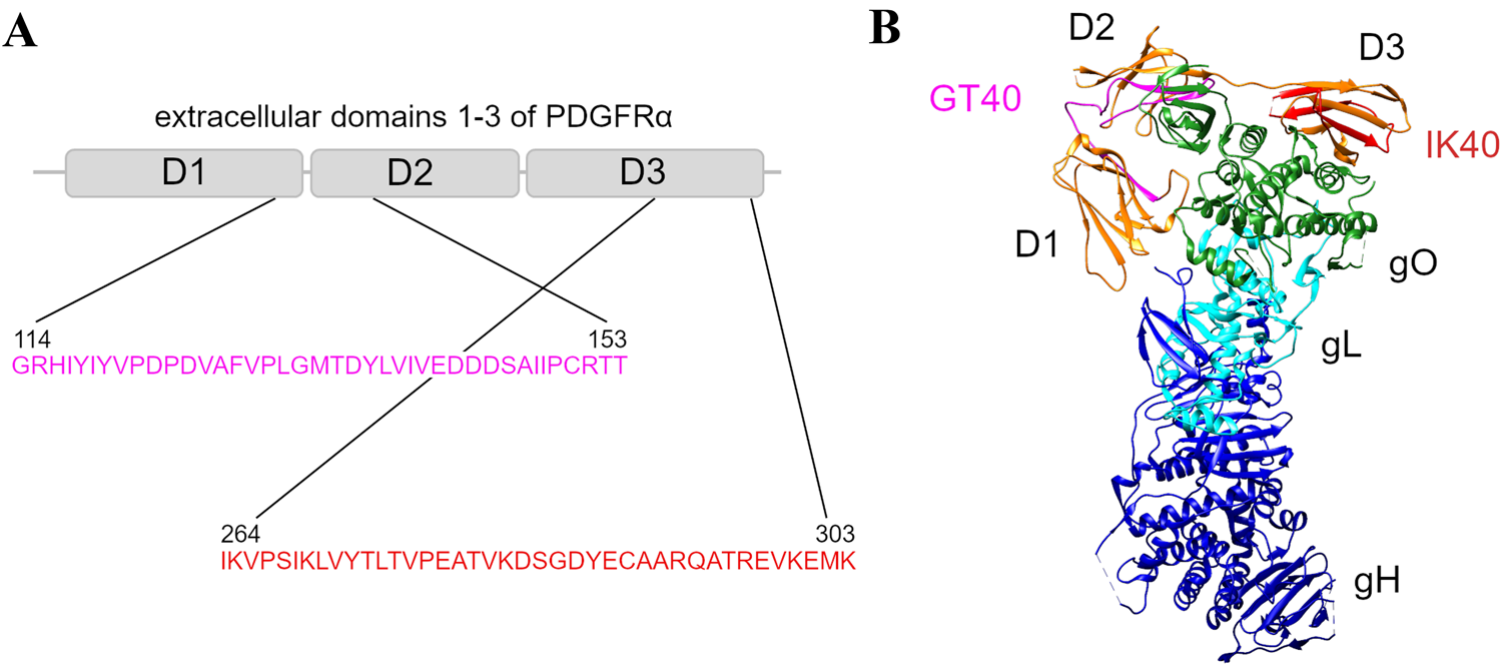
GT40 and IK40 are 40-mer peptides derived from the extracellular domain of PDGFRα. **A** Schematic organization of domains 1–3 of human PDGFRα including the localization and amino acid sequences of the HCMV inhibitory peptides GT40 and IK40. **B** Visualization of the trimeric gH/gL/gO complex of HCMV bound to PDGFRα domains 1–3 [39]. Localizations of GT40 and IK40 in PDGFRα are highlighted

**Fig. 5:**
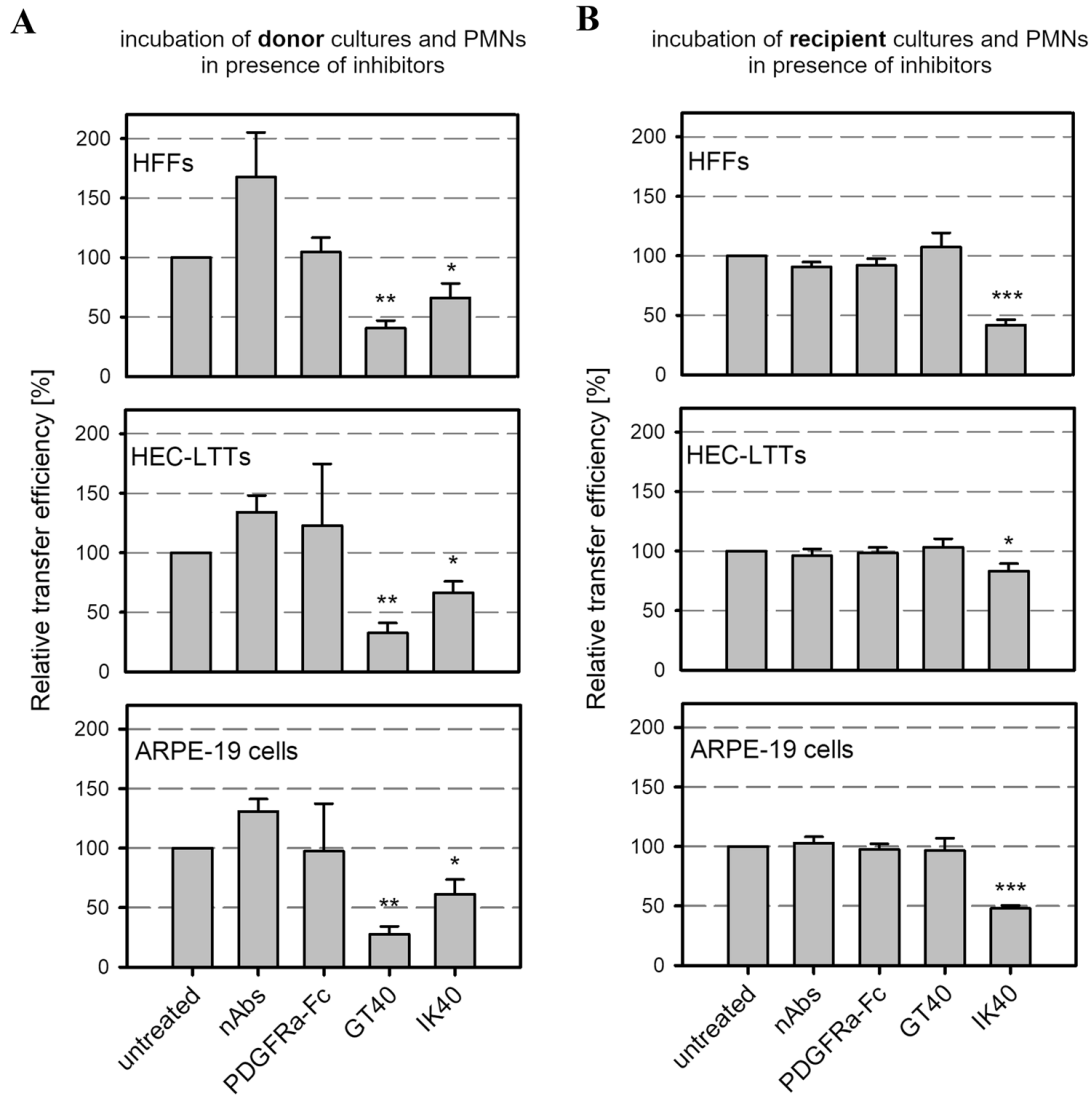
The PDGFRα-derived peptides GT40 and IK40 inhibit PMN-mediated transmission of HCMV. PMNs isolated from fresh blood samples of HCMV-seronegative donors were added to HFFs that were infected with 5 different clinical isolates or Merlin-pAL1502. After 3 h at 37 °C, PMNs were applied onto uninfected HFFs, HEC-LTTs or ARPE-19 cells. After 3 h, PMNs were removed from the recipient cell cultures. To evaluate their effect on PMN-mediated spread, HCMV entry inhibitors were included during incubation of PMNs with donor cultures (**A**) or recipient cultures (**B**). nAbs, a soluble PDGFRα-Fc chimera or PDGFRα-derived peptides (GT40, IK40) were added at 0.5 mg/ml, 120 ng/ml or 0.45 mg/ml, respectively. Cells were fixed on the next day and stained via indirect immunofluorescence for viral IE Ag. Cell nuclei were counterstained with DAPI. Transfer efficiencies in the presence of inhibitors were calculated and are shown relative to the transfer efficiency of untreated cells. Error bars represent the standard error of the mean of 3 (**A**) or 11 (**B**) individual experiments, asterisks indicate significant differences as compared to untreated transfer (*p value < 0.05; **p value < 0.01; ***p value < 0.001)

**Fig. 6:**
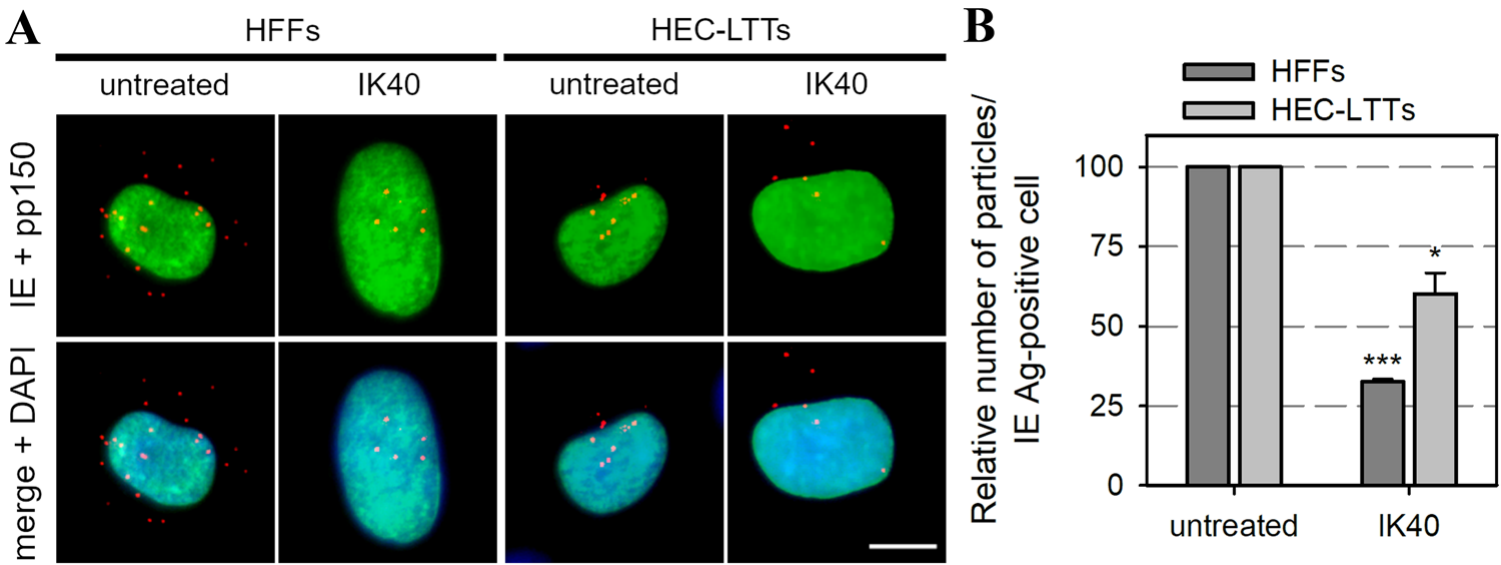
The PDGFRα-derived peptide IK40 reduces the number of transferred virus particles. PMN-mediated transfer was performed with cell-associated HCMV isolates onto HFFs and HEC-LTTs in presence or absence of the inhibitory peptide IK40 (0.45 mg/ml) during incubation of PMNs and recipient cells. **A** The recipient cells were fixed on the next day and stained via indirect immunofluorescence for viral IE Ag (green nuclei). Capsid-associated tegument protein pp150 was stained for detection of virus particles (red dot-like signals). Cell nuclei were counterstained with DAPI (blue nuclei). Scale bar = 10 µm. **B** The number of virus particles per infected cell in presence of IK40 was determined and is shown relative to the number of virus particles on untreated cells (70 cells per condition). Error bars represent the standard error of the mean of 3 individual experiments, asterisks indicate significant differences as compared to untreated transfer (*p value < 0.05; ***p value < 0.001)

The exact mechanism of virus-transfer via PMNs is unclear. It is, however, conceivable that viral particles are exposed at the surface of PMNs before they are transmitted to recipient cells and can thus be targeted by inhibitors with neutralizing capacity. Hence, we were curious whether the transfer of cell-associated isolates from PMNs to various recipient cell types can be targeted by antiviral agents such as nAbs, a soluble PDGFRα-Fc chimera or two PDGFRα-derived 40-mer peptides. Both peptides, GT40 and IK40, are located in the extracellular domain of PDGFRα (Fig. 4) and were recently described to inhibit cell-free HCMV infection [29]. To evaluate the effects of those inhibitors on PMN-mediated transmission of clinical isolates, we performed transfer experiments as described before but included the inhibitors either during the incubation of yet uninfected PMNs with the donor cultures (“uptake”) or during the incubation of infected PMNs with the different recipient cell types (“transfer”). The next day, viral IE Ag was detected via immunofluorescence and nuclei were counterstained with DAPI. For each treatment, the transfer efficiency was calculated as the ratio of cells positive for viral IE Ag to the total cell count and finally normalized to untreated PMN-transfer. Neither HCMV-specific hyperimmunoglobulin, containing nAbs, nor PDGFRα-Fc inhibited the transmission of cell-associated HCMV via PMNs, regardless of the step of transfer at which they were added. In contrast, the PDGFRα-derived peptide GT40 significantly reduced PMN-mediated transmission of HCMV to HFFs, HEC-LTTs and ARPE-19 cells by 60–70% when applied during the uptake step (*p* values < 0.01) (Fig. 5A), whereas it was ineffective when added during the transfer step (Fig. 5B). IK40, unlike GT40, could affect both steps: when added during the uptake step, it inhibited transmission by about 40% (*p* values < 0.05), and it was also effective when applied during the transfer step, where it reduced the transfer efficiency on HEC-LTTs by 20% (*p* value < 0.05) and 50–60% on ARPE-19 cells and HFFs (*p* values < 0.001). To further investigate the mode of action of IK40, which was the only agent to inhibit both steps of transmission, experiments in presence of the peptide during incubation of already infected PMNs and recipient cells were repeated on endothelial cells and fibroblasts. As the inhibitory effect of this peptide is assumed to depend on steps of the HCMV entry process [29], we wanted to evaluate whether IK40 interferes with the number of transferred viral particles. For that, we fixed the recipient cultures 1d after the transfer and visualized the highly abundant capsid-associated tegument protein pp150 via immunofluorescence staining, which results in dot-like signals that represent individual virus particles [29, 38, 42]. In addition, we stained for viral IE Ag, and nuclei were counterstained with DAPI. 70 cells per treatment and cell type were evaluated regarding the number of pp150 signals per nucleus positive for IE Ag. In comparison to untreated transfer, IK40 reduced the mean numbers of HCMV particles per infected recipient cell from 25 to 8 on HFFs and from 12 to 7 particles on HEC-LTTs (Fig. 6A), corresponding to a significant decrease by 70% (*p* value < 0.001) and 40% (*p* value < 0.05), respectively (Fig. 6B). Again, the inhibitory effect of IK40 was more pronounced in fibroblasts, providing a plausible explanation for the cell type-dependent differences concerning the transfer of infection (Fig. 5B). In summary, these experiments demonstrated that it is possible to interfere with the transfer of recent HCMV isolates via PMNs.

## Discussion

The data obtained in this study provide formal evidence that PMNs can transfer cell-associated HCMV isolates from infected fibroblast cultures to various uninfected cultures. This cell culture model closely mimics HCMV transfer via PMNs as it likely occurs in vivo and can be used for a variety of approaches in both basic and translational HCMV research.

Blood cells of the myeloid lineage are considered vehicles for hematogenous dissemination of HCMV, based on the findings that leukocyte depletion prevents transfusion-associated viral transmission and that infectious virus has been isolated from monocytes and PMNs of HCMV-infected patients [8, 9, 11, 43]. Among the different leukocyte subpopulations, PMNs were the major source of infectious virus [44, 45] and carried the highest relative amount of HCMV genome copies [15]. The concept that PMNs can act as vehicles to spread HCMV infection is supported by cell culture data. Repeatedly, uptake of HCMV from infected monolayers by PMNs has been reported using nuclear pp65 signals as a readout that mimics the in vivo “antigenemia” assay [18–21], and one report also mentioned transfer of infectious virus by such pp65-positive PMNs to uninfected monolayers [22]. Our data confirm these findings and go further by showing that PMNs can take up pp65 from fibroblasts that do not release infectious progeny but spread HCMV in a strictly cell-associated manner, and that such PMN preparations can transmit virus to other cell cultures. Together with our observation that nAbs cannot inhibit this transmission mode, it is reasonable to assume that cell-associated HCMV can exploit this transmission route in vivo as a means of immune evasion.

Assuming a central role for PMN-mediated spread in the pathogenesis of systemic HCMV infections, it is tempting to speculate on viral or host factors that might modulate the efficiency of this dissemination mode. However, our transfer experiments, conducted with five different clinical isolates and PMNs from four different donors, did not reveal striking differences between different isolates or donors. Rather, the transfer efficiencies were remarkably consistent. Hence, the differences we observed between the recipient cultures appear all the more noteworthy. The highest transfer rates were found with endothelial cells followed by fibroblasts, and both cell types differed significantly from epithelial cells that showed the lowest transfer rate. The particular susceptibility of endothelial cells could provide a benefit for the virus, as these cells form the inner cell layer of vessel walls and their infection may be the starting point for virus entry into the surrounding tissues.

Strategies to block this spreading route could be a useful complement to currently available treatment options. Interestingly, nAbs and lactoferrin were reported to inhibit virus uptake by PMNs from infected endothelial cells [21]. However, the virus strain used in these assays presumably releases cell-free progeny and hence an effect of these inhibitors on free virus cannot be excluded. In our experiments with strictly cell-associated HCMV isolates, nAbs were unable to block virus uptake by PMNs or subsequent transfer to recipient cultures, which is consistent with their failure to block cell-to-cell-spread in fibroblast cultures [33, 35]. Likewise, PDGFRα-Fc, a soluble decoy HCMV receptor that resembles nAbs regarding its mode of action [29], failed to reduce the uptake of isolates to PMNs or the transfer of infected PMNs to the various recipient cultures. One explanation is that the virus is transferred from cell-to-cell at zones of close cell–cell contacts that limit the access for large molecules like antibodies or PDGFRα-Fc. This is supported by remarks that PMN-mediated transfer of HCMV depended on cell–cell contacts and that it was reduced by antibodies against the intercellular cell-adhesion molecule 1 [19, 22], unfortunately without showing corresponding data. In line with this hypothesis, the PDGFRα-derived peptide IK40, which is 40- to 50-fold smaller than PDGFRα-Fc or nAbs, significantly inhibited PMN-mediated transmission to fibroblasts, endothelial and epithelial cells, irrespective of whether it was added during uptake or transfer. While this is consistent with the idea of size exclusion at the sites of HCMV uptake from infected cells to PMNs and subsequent transfer from PMNs to recipient cells, the failure of the GT40 peptide during incubation of already infected PMNs and the recipient cells remains unexplained. Both peptides are localized at positions in the extracellular domain of PDGFRα that appear to be involved in the interaction with HCMV glycoprotein O, and it is noteworthy that they contain the key amino acids M133, L137, I139 (GT40), and K265 (IK40) of these interaction sites [39]. Obviously, these peptides, although derived from the same entry inhibitor, differ in their mode of action, which should be addressed in future analyses. As for the effect of IK40, the number of transferred virus particles was reduced in the presence of this peptide, and the degree of reduction corresponded well with its inhibitory effect in fibroblasts and endothelial recipient cultures. Since this PDGFRα-derivative is assumed to act on virus entry, this finding favors the idea of a local virus release at contact sites between PMNs and recipient cells, sometimes described as virological synapses [46]. Alternatively, microfusion events between infected endothelial cells and PMNs have been proposed [19], which could allow transfer of subviral particles. If such microfusions are also formed during contact of infected PMNs with uninfected endothelial cells, a combination of both mechanisms, local release of virus progeny and direct transfer of subviral particles, could explain why the inhibition by IK40 is not complete. In summary, although open questions remain regarding the precise mode of action, GT40 and IK40 provide proof of principle that PMN-mediated hematogenous dissemination can be targeted for therapeutic purposes.

Beyond the issue of PMN-mediated dissemination, use of PMNs as vehicles can more generally facilitate research with recent clinical isolates, including the analysis of cell tropism after transfer to endothelial or epithelial cells. Compared to previously published procedures based on coculture with terminally differentiated fibroblasts [26] or direct isolation in endothelial or epithelial cells [47, 48], PMN-mediated transfer of isolates initially propagated in fibroblast cultures appears less demanding and more reliable. Our data furthermore show that isolates continue to grow in a strictly cell-associated mode after transfer in recipient cultures. We assume that early transfer to endothelial or epithelial cells may reduce or even abrogate the selective pressure on the viral UL128 locus that has been associated both with the extended cell tropism of recent isolates and their cell-associated phenotype [23, 27].

In summary, we proved that PMNs can transfer strictly cell-associated HCMV isolates to a variety of cell types, emphasizing their role as a central key player in the hematogenous dissemination of HCMV and we provided proof of principle that this dissemination route can be targeted by inhibitors.
